# Psychometric properties of the Chinese version of adjustment disorder new module-20 in breast cancer patients

**DOI:** 10.1186/s40359-020-00494-2

**Published:** 2020-12-09

**Authors:** Haiyan Tang, Huihua Xiong, Lingchao Deng, Andreas Maercker, Jun Zhang, Heng Meng

**Affiliations:** 1 Department of Maternal and Child Health, School of Public Health, Tongji Medical College, Huazhong University of Science & Technology, 13 Hangkong Road, Qiaoko District, Wuhan, Hubei 430030 People’s Republic of China; 2 Department of Comprehensive Oncology, Tongji Hospital, Tongji Medical College, Huazhong University of Science & Technology, Wuhan, People’s Republic of China; 3 Department of Psychology, University of Zurich, Zurich, Switzerland; 4 Wuhan Mental Health Center, Wuhan, People’s Republic of China

**Keywords:** Adjustment disorder, Adjustment Disorder New Module-20(ADNM-20), Self-rating scale, Psychometric properties, Factorial validity, Reliability

## Abstract

**Background:**

After the new definition of adjustment disorder (AjD) by the International Classification of Diseases-11(ICD-11), AjD has attracted more and more attention. Adjustment disorder new module-20 (ADNM-20), which is used to diagnose AjD, has been verified in some countries, but it has not been verified in China. As a result, the purpose of this study was to investigate the validity and reliability of the Chinese version of the Adjustment disorder new module-20 (ADNM-20) in female breast cancer patients.

**Methods:**

The ADNM-20 translated into Chinese employed the translation and back translation technique. Three hundred fifty four newly diagnosed (< 1 year) female breast cancer patients were recruited from Tongji Hospital and Hubei Cancer Hospital in Hubei, China. The patients completed the self-report questionnaire including demographic characteristics and the scale ADNM-20. Data on psychometric properties were evaluated in terms of internal consistency, item-total correlations, test-retest reliability, and factorial validity.

**Results:**

ADNM-20 core symptoms included 8 items and two factors, which were extracted by using exploratory factor analysis (EFA). It could explain 61.74% of the total variance. ADNM-20 accessory symptoms including 12 items and four factors, which were extracted by using EFA. It could explain 68.34% of the total variance. Cronbach’s α coefficient for ADNM-20 was 0.93, split-half reliability was 0.87, and the test-retest correlation coefficient was 0.74. The correlation coefficient between each subscale was ranged from 0.53 to 0.71 (*P* < 0.01), while the correlation coefficient between the subscales and total scale was ranged from 0.79 to 0.89 (*P* < 0.01).

**Conclusions:**

The study verified the validity and reliability of the Chinese version of ADNM-20. It is applicable to measure the prevalence of adjustment disorder in the breast cancer population.

## Background

Adjustment disorder (AjD) is a common mental disorder characterized by a series of psychological behavioral responses caused by inadequate adaptation to stressors [1]. Previous surveys of 4,887 psychiatrists worldwide showed above 50% of them performed at least one AjD diagnosis every week [2, 3]. AjD is also a common comorbidity in cancer patients. A meta-analysis indicated that the prevalence of AjD in cancer patients was 15.4% [4]. Hund et al. found that except head and neck cancer, breast cancer had the highest prevalence of AjD was than other cancer, so this group should receive more attention [5]. Besides, the 5-year and 10-year survival rates of breast cancer were 87 and 82%, respectively [6].

China accounts for 12.2% of all newly diagnosed breast cancer cases globally, 9.6% of all breast cancer deaths, and is on the rise compared with the past [7]. However, to our knowledge most of the studies conducted in China on breast cancer were focused on adolescents, college students, soldiers and other groups, studies assessing AjD in breast cancer are scarce. For instance, a study by Liu Jing found that the prevalence of AjD in recruits was 3.33% [8]. Consequently, we should attach importance to their quality of life including mental health.

The common scales used to diagnose AjD in the past included the Structured Clinical Interview for DSM-IV(SCID), MINI-International Neuropsychiatric Interview 5.5 etc. Nonetheless, in 2018, the 11th edition of the International Classification of Diseases (ICD-11) has newly defined AjD, and put it in the same grouping as post-traumatic stress disorder (PTSD) and complex post-traumatic stress disorder [9]. According to the new definition, diagnostic criteria for AjD contain identifiable stress events, preoccupation and failure to adapt symptoms, as well as damaged important functional areas such as family, social interaction and education that result from inadequate adaptation to stressors. However, based on different symptoms, AjD can be accompanied by accessory symptoms of depression, anxiety, avoidance and impulsivity. Previous scales for AjD cannot completely meet the requirements of the above diagnostic criteria. Maercker et al. [10] developed Adjustment Disorder New Moudle (ADNM) to diagnose AjD. During the construction of the scale, a team of experienced clinicians in Germany drafted a library of 55 symptoms covering preoccupation, failure to adapt, and affiliated symptoms.

Finally, ADNM-20 was generated by refining ADNM based on the corresponding professional knowledge and research results. Studies on Einsle and Dobricki have shown that ADNM-20 has good psychometric characteristics [10, 11] and is consistent with the definition of AjD in ICD-11. Previous studies have proposed a total of 3 model structures for ADNM-20 [12]. The study of Einsle supported the 6-factor structure of the 20 symptom items of the scale: failure to adapt, preoccupation, anxiety, depression, impulsivity, and avoidance [10]. The study of Lorenz proposed a unidimensional structure of ADNM-20 [13]. Since ICD-11 only focuses on the core symptoms of “failure to adapt”, and “preoccupation”, Zelviene et al. proposed a 2-core factor model [14]. Additionally, ADNM-20 has been translated into German [15] and Lithuanian [14]. While the Chinese Classification of Mental Disorders and Diagnostic criteria-3 (CCMD-3) put AjD into the classification of stress-related disorders [16]. The definition of AjD in CCMD-3 is roughly the same as in ICD-10. There is a lack of scales to diagnose AjD consistent with the new definition of ICD-11 in China. Besides, previous studies on AjD have focused on non-clinical samples, such as the sample of involuntary job loss [17], traumatic attacks [18] or soldiers [19], but less clinical samples. Thus, to fill this gap, we conducted a study on AjD among female breast cancer patients to analyze the reliability and factorial validity of the Chinese version of ADNM-20, providing theoretical basis and support for the use and promotion of the scale in China and the applicability of clinical samples.

## Methods

### Chinese translation

The ADNM-20 translated into Chinese employed the translation and back translation technique [12]. Firstly, the ADNM-20 was translated from English to Chinese by a bilingual technical writer, and back-translated by another bilingual writer. Secondly, two other experts who are experienced in mental health research independently reviewed the initial forward and backward translations. Finally, discrepancies between the translations were discussed by the above two experts and corrections were made on mutual agreement, and the Chinese version of ADNM-20 was generated according to the suggestions of them [20].

### Participants

Participants were recruited from Tongji Hospital and Hubei Cancer Hospital in Hubei Province, China, from July 2018 to May 2019 by convenience sampling. According to the size of the tumor and lymph node or distant metastasis, breast cancer is divided into stages 0, I, II, III and IV. Considering that breast cancer patients with stage IV need more complex treatment and may have cognitive dysfunction [21], only patients with 0, I, II and III were included in this study. Besides, we put patients with stage 0 or I breast cancer in the same group based on the similar severity of stage 0 or I breast cancer. The inclusion criteria including a diagnosis of breast cancer within 1 year (including patients in active treatment and completed the treatment process), female aged more than 18 years, ability to read and write simple Chinese characters, clear consciousness and cognition (be able to accurately answer questions on persons, place, and time within 30s), and willingness to complete questionnaire independently. The exclusion criteria including intellectual and/or cognitive impairments, and have other active cancers.

The sample size for validity and reliability analysis of the scale should be 10 to 15 times the number of items, and there are 20 items in ADNM-20, so the sample size of this study should be at least 200 [22].A total of 400 patients were recruited. Out 400 of patients, 354 patients agreed to participate in this study. The age of the participants was ranging from 25 to 79, the response rate was 82.90% and 50 patients were randomly retested within 1 month.

### Measures

#### Instruments

ADNM-20 consists of a stressor list and a symptom list. The stressor list was the stress events that ICD-10 identified in 1992 that may trigger AjD. It contains seven acute stress events (e.g. divorce, moving house) and nine chronic stress events (e.g. conflict with neighbors, serious illness). Respondents were asked to select all the stressful events they had experienced in the past 2 years, fill in the occurrence time of acute events and the occurrence and end time of chronic events. The respondents then specified the top three events that caused them subjective distress. For the following calculations, we assumed that the magnitude of the influence of the severe events would be ranged from high to low in order the sequence of the events which were mentioned. In addition, the participants completed the second part of the list of AjD symptoms accordingly.

The symptom list of ADNM-20 consists of 19 symptom items plus 1 item that measures functional impairment. The scale measured two core symptoms: failure to adapt and preoccupation. Four accessory symptoms were also measured: anxiety, impulsivity, avoidance, and depression. The frequency of all symptoms were assessed with a 4-point Likert scale (1 = never; 2 = less; 3 = sometimes; 4 = often), the higher is the score, the more serious the symptoms.

#### Procedure

Trained investigators conducted face-to-face questionnaire survey on the patients in the hospital, and the patients filled the questionnaire by themselves. If a patient was unable to fill the questionnaire due to physical conditions, the investigator helped him to fill out the questionnaire. The investigator stated only the corresponding questions without any leading words. Before the questionnaire survey, a written informed consent was signed by patients or by their family members, and the survey was approved by the Ethics Committee of Tongji Medical College of Huazhong University of Science and Technology, China.

#### Statistical analysis

EpiData3.1 was used for parallel and double-entry of the questionnaire to ensure the quality of the questionnaire entry. SPSS 22.0 version was used to analyze data. Descriptive statistics were conducted for participant features and for the score of ADNM-20. Through skewness and kurtosis analysis, it was found that the skewness of the total items of ADNM-20 was − 0.14, and the kurtosis was − 0.50, which tended to be normally distributed. Therefore, the variance analysis and independent sample t-test were used to analyze data.

Exploratory factor analysis (EFA) was utilized to evaluate the structural validity of the questionnaire. Principal Axis Factoring (PAF) and rotation method (Promax) were used to explore the structure of ADNM-20 in the EFA. Confirmatory factor analysis (CFA) was conducted by maximum likelihood (ML) method to test the construct validity of ADNM-20. And the CFA was carried out by using AMOS 24.0 version. The internal consistency reliability of the questionnaire was evaluated by Cronbach’s α coefficient, and the split-half reliability was evaluated by using the Guttman Split-Half coefficient. Pearson correlation was used to assess the item-total correlations and intraclass correlation coefficient (ICC) was calculated to evaluate the test-retest reliability [23].

## Results

### Demographic characteristics and the score of ADNM-20

As shown in Table 1, patients’ ages ranged from 25 to 79 years (Mean, 47.2; SD, 10.2). In terms of occupation, 42.94% were housewives. Breast cancer stage I was accounted for 33.62%, breast cancer stage II was accounted for 35.59%, and breast cancer stage III was accounted for 30.79%. While 11.87% of patients wanted to have children before developing cancer.

**Table 1:** Demographic characteristics and the score of ADNM-20

Variable	*n* (%)	ADNM-20 score ((M ± SD)	*F*/t
Age			1.36
25 ~ 35	45 (12.97)	22 ~ 78 (51.16 ± 12.81)	
35 ~ 45	110 (31.70)	20 ~ 77 (48.90 ± 11.27)	
45 ~ 55	125 (36.02)	19 ~ 80 (49.26 ± 13.53)	
55 ~ 65	45 (12.97)	24 ~ 79 (50.04 ± 11.21)	
≥ 65	22 (6.34)	21 ~ 67 (43.82 ± 14.20)	
Education			0.16
Primary and below	69 (19.88)	26 ~ 80 (49.71 ± 11.14)	
Junior high school	112 (32.28)	22 ~ 71 (48.62 ± 12.11)	
High school	70 (20.17)	19 ~ 79 (48.77 ± 13.09)	
University and above	96 (27.67)	20 ~ 78 (48.46 ± 13.14)	
Occupation			5.99^**^
White-collar worker	81 (22.88)	20 ~ 75 (46.82 ± 13.23)	
Blue-collar workers	52 (14.69)	24 ~ 71 (47.65 ± 11.65)	
Housewife	152 (42.94)	22 ~ 80 (52.22 ± 11.55)	
Other	69 (19.49)	19 ~ 79 (45.94 ± 13.06)	
Clinical stage			0.77
Stage 0 or I	119 (33.62)	20 ~ 80 (49.56 ± 12.14)	
Stage II	126 (35.59)	19 ~ 72 (49.71 ± 12.49)	
Stage III	109 (30.79)	20 ~ 79 (47.85 ± 13.00)	
Want to give birth before illness			2.24^*^
Yes	40 (11.87)	22 ~ 72 (53.08 ± 11.31)	
No	297 (88.13)	19 ~ 80 (48.40 ± 12.53)	

Notes:^*^P < 0.05, ^**^P < 0.01

The score of ADNM-20 was different through different occupations (*P* < 0.01). Housewife’s score (52.22 ± 11.55) was higher than other groups. There was a significant difference of the score between the patients who wanted to have a baby before cancer diagnosis and patients who did not, while the score of the former was higher than the latter (*P* < 0.05). There was no significant difference between the scores of other variables.

### Incidence of stress life events

All patients suffered from stressful life events in the past 2 years. Except breast cancer, the incidence of economic pressure was highest (49.71%), followed by the workload problem (29.94%) and no leisure activities (28.25%) (Table 2). While 70.90% of patients reported that the most stressful event was breast cancer, 11.30% of patients reported economic pressure, 4.20% reported workload problem, 4.20% of patients reported family conflicts, and 3.10% of patients reported lover’s health.

**Table 2:** Lifetime prevalence rates of severe life events

Life events	*n*	Rates (%)
Acute events		
Death of a loved one	9	2.54
Moving	29	8.19
Divorce	15	4.24
Termination of leisure activity	100	28.25
Retirement	29	8.19
Accident	12	3.39
Criminal act (e.g. burglary)	6	1.69
Chronic stressors		
Financial difficulties	176	49.71
Too much or too little work	106	29.94
Family conflicts	36	10.17
Illness/care for a loved one	92	25.99
Unemployment	60	16.95
Pressure to meet deadlines	72	20.34
Serious illness	354	100
Conflicts at job	17	4.8
Conflicts with neighbors	3	0.85
Other	30	8.47

### Factorial validity

The fixed factor number of core symptom factor analysis was two, and the factor loading value of each item was more than 0.55. Exploratory analysis showed that Kaiser-Meyer-Olkin (KMO) value was 0.88, Bartlett spherical test was significant (*P* < 0.01), indicating that the item-pool was suitable for a factorial validation. Explanatory factor analysis (EFA) with Principal Axis Factoring pointed to a two-factor solution, which can explain 61.74% of the variance. Combined with the content of scale items, factor 1 is “failure to adapt” and factor 2 is “preoccupation”, details are shown in Table 3.

**Table 3:** Factors and factor loading of ADNM-20 core symptom scale

Factor 1		Factor 2	
Item	Loading	Item	Loading
17	0.66	4	0.81
20	0.62	2	0.79
10	0.57	15	0.64
19	0.55	13	0.62

The same method was used to analyze the items of ADNM-20 accessory symptoms. The number of fixed factors was four and the load of each item was more than 0.50. Explanatory analysis showed that the KMO value was 0.90, Bartlett spherical test was significant (*P* < 0.01), indicating that the item-pool was suitable for a factorial validation. Four factors were obtained, which explained 68.34% of the variance. Combined with the contents of scale items, factor 1 was named “anxiety”, factor 2 was named “impulsivity”, factor 3 was identified as “avoidance”, and factor 4 was identified as “depression” (Table 4).

**Table 4:** Factors and factor loads of ADNM-20 symptom subscale

Factor 1		Factor 2		Factor 3		Factor 4	
Item	Loading	Item	Loading	Item	Loading	Item	Loading
16	0.78	12	0.94	7	0.73	1	0.77
6	0.70	9	0.83	11	0.66	18	0.62
		8	0.50	3	0.60		
				14	0.49		

To further determine the factor structure of the ADNM-20, CFA was conducted. After adjustment, the CFA of the model resulted in *x*^2^/df = 4.10, CFI = 0.85, TLI = 0.80, IFI = 0.86, NFI = 0.82, and RMSEA = 0.093 (90% CI, 0.085–0.100), indicating that the fitting of the model is acceptable. (CFI: comparative fit index; TLI: Tucker–Lewis index; IFI: incremental fit index; NFI: normed fit index; RSRMR: root mean square error of approximation.)

### Reliability

The internal consistency analysis of the total ADNM-20 scale showed that the Cronbach α coefficient was 0.93, the split-half reliability was 0.87 and the ICC was 0.74 (Table 5).

**Table 5:** Internal consistency and retest reliability of ADNM-20

	Cronbach α	Split-half reliability	Pearson *r*
Failure to adapt	0.69	0.74	0.57^**^
Preoccupation	0.82	0.65	0.64^**^
Anxiety	0.67	0.67	0.52^**^
Impulsivity	0.80	0.36	0.63^**^
Avoidance	0.73	0.75	0.38^*^
Depression	0.61	0.62	0.61^**^
Total scale	0.93	0.87	0.74^**^

Notes: ^*^P < 0.05, ^**^P < 0.01

The correlation between the total scale and the six subscales of “failure to adapt”, “preoccupation”, “anxiety”, “impulsivity”, “avoidance” and “depression” were 0.83, 0.89, 0.84, 0.82, 0.79 and 0.82, respectively (*P* < 0.01). The correlation of each subscale was between 0.53 and 0.71 (*P* < 0.01). While the correlation between each subscale was lower than between each scale and the total scale (Table 6).

**Table 6:** Correlation between the subscales and the total table

	Failure to adapt	Preoccupation	Anxiety	Impulsivity	Avoidance	Depression
Failure to adapt	1.00					
Preoccupation	0.68	1.00				
Anxiety	0.68	0.71	1.00			
Impulsivity	0.57	0.68	0.66	1.00		
Avoidance	0.53	0.60	0.61	0.57	1.00	
Depression	0.67	0.76	0.62	0.65	0.54	1.00
Total scale	0.83	0.89	0.84	0.82	0.79	0.82

## Discussion

The prevalence of AjD found by Zelviene et al. [1] in the general population was from 1.00–2.00%. In a transnational study conducted in Finland, Ireland, Norway, and Spain, the prevalence of AjD found in the general population diagnosed by the ICD-10 and clinical assessment of neuropsychiatry was from 0.20–1.00% [24]. Furthermore, the prevalence of AjD found in clinical and high-risk samples was higher than what found in the general population [1]. A primary health care study conducted in Spain showed that 2.90% of participants were affected by AjD [25]. As reported in an Australia longitudinal study, the prevalence of AjD among people suffering at 3 months and 12 months of follow-up were 18.9 and 16.3% respectively [1]. Based on 70 studies, cancer patients were more susceptible to AjD than was the general population, while the prevalence of AjD in patients who underwent oncology or blood tests was 19.4%, compared with 14.9% for major depressive disorder and 16.3% for depression [13, 26, 27]. Therefore, it is imperative to pay attention to AjD. Although AjD has a high prevalence in health care, there is a lack of study to focus on it. We found 139,979 research projects in the past 10 years yielded by PubMed using “depressive disorder” as the key word. In contrast, only 401 studies were searched out in the past 10 years with “adjustment disorder” as the key word [1]. What’s more, AjD is not the item of national major health surveys, which leads to limited epidemiological information about the prevalence of AjD [28]. This is related to the lack of a reliable assessment tool for AjD. Currently, we diagnosed AjD with measurements established for other diseases and disorders, such as Life-BREF Scale for Diagnosing AjD in DSM-V [29], the Hamilton Anxiety Scale, Shihan Disability Scale, Montgomery-Asperger Depression Scale for AjD diagnosis in DSM-IV [30]. The ADNM-20 developed by Maercker [15, 31] met the new requirements for AjD diagnosis and agreements with the definition of ICD-11.

The primary criterion for the diagnosis of AjD is the occurrence of stress events. The subjects of this study were women who had been diagnosed with breast cancer within 1 year. 70.90% of them reported that breast cancer was the most stressful event. Besides, 11.30 and 4.20% of them indicated that financial difficulties and job-related events were the most stressful events, respectively. It could be seen that, in addition to breast cancer which could bring great pressure to women, work, financial problems are not to be underestimated [6]. The two may also interact each other, and the financial problems can be caused by the inability to work and the treatment after the illness, which will lead patients in a more embarrassing state and increasing prevalence of AjD.

Exploratory factor analysis showed that the two core symptom subscales of ADNM-20 (preoccupation and failure to adapt) and the three subscales of accessory symptom (anxiety, avoidance, and impulsivity) had good factorial validity. The result of factor analysis of the depression scale was different from the theoretical structure. Item 5 of the original depression scale was eliminated because the factor loading was less than 0.45. Previous studies have also shown that ADNM-20 was more effective in identifying the core symptoms of AjD, and the diagnosis of subtypes need to be further studied [10]. And CFA showed good factorial validity of ADNM-20.

ADNM-20 has a good internal consistency in this study, and Cronbach α was 0.93. All subscales had good reliability, and Cronbach α was between 0.61 and 0.82. The preoccupation and impulsivity subscales have the best internal consistency, and the worst was the depression subscale. In the related research, the Cronbach α of ADNM-20 was between 0.74 and 0.90 [10], and in some studies, the Cronbach α was between 0.81 and 0.85 [32]. A study investigating theft victims showed that the internal consistency of the ADNM-20 was very high, the Cronbach α was 0.94, and the internal consistency of the ADNM 20 subscales was ranged from 0.80 to 0.89 [33]. The Cronbach α of the Lithuanian version of the ADNM-20 was ranged from 0.65 to 0.87 [14]. ADNM-20 had a good ICC (0.74), the highest ICC of each subscale was preoccupation (0.64), and the lowest was avoidance (0.38). Perhaps because the retest of this study was completed within 1 month, with time, the degree of avoidance of the disease decreased. The retest time should be shortened within 2 weeks, which is also the deficiency of this study, which will be improved in the future study.

The correlation coefficient between each subscale and the total scale was between 0.79 and 0.89, the highest correlation was the preoccupation subscale, and the lowest was the avoidance subscale. The correlation between the subscales was between 0.53 and 0.71, the best correlation was the preoccupation and depression subscale, while the worst was the failure to adapt and avoidance subscale. Besides, the correlation between each subscale was lower than between the subscale and the total scale, which further showed that the scale had good convergence validity. Other related studies also showed that there was a moderate correlation between the subscales of ADNM-20 and the total scale [10].

Therefore, ADNM-20 had good psychological measurement characteristics in research, and it was easier to operate than other scales. Non-psychiatrist doctors in hospitals can also use ADNM-20 to screen patients suffering from AjD. Community doctors can also use the scale to screen and diagnose community service objects. Because of its high performance-to-price ratio and convenient self-evaluation operation form, it is suitable for large sample research and high research and utilization value.

### Limitation

However, there are still some limitations in this study: First, the subjects covered only female breast cancer patients, not male patients and other groups. The age of all participants was over 25 years old, those under 25 years old were not studied. Therefore, the applicability in men, other groups and small age groups needs to be further studied. Second, the subjects were only selected in two general hospitals in Wuhan, which was not representative enough, so it was still necessary to supplement the objects in other areas, expand the sample size and do further confirmative research. At last, since there is no good scale for the diagnosis of AjD before, and the only gold standard is the diagnosis of clinical experts, this study verified only the factorial validity of ADNM-20, but not the criterion validity. And we did not conduct a structured interview based on the new ICD-11 criteria. It would not be able to determine the sensitivity and specificity of the ADNM-20. The future research will make up for the above deficiency.

## Conclusion

ADNM-20 had good validity and reliability in measuring the prevalence of AjD in female breast cancer patients. The factor analysis results of the scale were consistent with the theoretical structure. The internal consistency coefficient Cronbach α and the retest correlation coefficient of the total scale were high, and the results were under the characteristics of psychological measurement. ADNM-20 could be used to screen and diagnose AjD in breast cancer patients.

## Data Availability

The datasets generated and/or analysed during the current study are not publicly available due to the privacy of breast cancer patients, but are available from the corresponding author on reasonable request.

## Abbreviations

AjD: Adjustment disorder
ICD: International Classification of Diseases
ADNM-20: Adjustment disorder new module-20
DSM: Diagnostic and Statistical Manual of Mental Disorders
SCID: Structured Clinical Interview for DSM-IV
PTSD: Post-traumatic stress disorder
CCMD-3: Chinese Classification of Mental Disorders and Diagnostic criteria − 3
EFA: Exploratory factor analysis
PCA: Principal component method
CFA: Confirmatory factor analysis
ICC: Intraclass correlation coefficient
KMO: Kaiser-Meyer-Olkin
CFI: Comparative fit index
TLI: Tucker–Lewis index
IFI: Incremental fit index
NFI: Normed fit index
RSRMR: Root mean square error of approximation
